# Characterization and Comparative Genomic Analysis of a Deep-Sea *Bacillus* Phage Reveal a Novel Genus

**DOI:** 10.3390/v15091919

**Published:** 2023-09-13

**Authors:** Yuan Chen, Tianyou Zhang, Qiliang Lai, Menghui Zhang, Meishun Yu, Runying Zeng, Min Jin

**Affiliations:** 1 State Key Laboratory Breeding Base of Marine Genetic Resource, Third Institute of Oceanography, Ministry of Natural Resources, Xiamen 361000, China; chenyuan@tio.org.cn (Y.C.); laiqiliang@tio.org.cn (Q.L.); menghlike@163.com (M.Z.); yumeishun@tio.org.cn (M.Y.); 2 Fujian Provincial Center for Disease Control and Prevention, Fuzhou 350000, China; 18003851933@163.com; 3 Southern Marine Science and Engineering Guangdong Laboratory, Zhuhai 519000, China

**Keywords:** *Bacillus* phage, *Bacillus tequilensis*, myophages, deep sea, novel genus

## Abstract

As the most abundant biological entities, viruses are the major players in marine ecosystems. However, our knowledge on virus diversity and virus–host interactions in the deep sea remains very limited. In this study, vB_BteM-A9Y, a novel bacteriophage infecting *Bacillus tequilensis*, was isolated from deep-sea sediments in the South China Sea. vB_BteM-A9Y has a hexametric head and a long, complex contractile tail, which are typical features of myophages. vB_BteM-A9Y initiated host lysis at 60 min post infection with a burst size of 75 PFU/cell. The phage genome comprises 38,634 base pairs and encodes 54 predicted open reading frames (ORFs), of which 27 ORFs can be functionally annotated by homology analysis. Interestingly, abundant ORFs involved in DNA damage repair were identified in the phage genome, suggesting that vB_BteM-A9Y encodes multiple pathways for DNA damage repair, which may help to maintain the stability of the host/phage genome. A BLASTn search of the whole genome sequence of vB_BteM-A9Y against the GenBank revealed no existing homolog. Consistently, a phylogenomic tree and proteome-based phylogenetic tree analysis showed that vB_BteM-A9Y formed a unique branch. Further comparative analysis of genomic nucleotide similarity and ORF homology of vB_BteM-A9Y with its mostly related phages showed that the intergenomic similarity between vB_BteM-A9Y and these phages was 0–33.2%. Collectively, based on the comprehensive morphological, phylogenetic, and comparative genomic analysis, we propose that vB_BteM-A9Y belongs to a novel genus under *Caudoviricetes*. Therefore, our study will increase our knowledge on deep-sea virus diversity and virus–host interactions, as well as expanding our knowledge on phage taxonomy.

## 1. Introduction

Viruses are the most abundant biological entities in the ocean. They are virtually present in all marine ecosystems. As estimated, there are an average of 10^7^ virus-like particles per milliliter of surface seawater, which makes viruses approximately an order of magnitude more abundant than prokaryotes. Despite their small size, viruses make up the second largest relative biomass in the ocean, exceeded only by the total biomass of prokaryotes [1]. The majority of marine viruses are phages that infect prokaryotes. Because of their enormous abundance and genetic diversity, viruses play a pivotal role in marine ecosystems. Viruses control host abundance and affect host community structures by lysing their hosts. Viruses also influence host diversity and evolution through horizontal gene transfer. Moreover, viruses affect local and global biogeochemical cycles not only by releasing substantial amounts of organic carbon and nutrients from host cells but also by assisting microbes in driving biogeochemical cycles with auxiliary metabolic genes (AMGs) [2,3,4,5]. For example, approximately 1028 viral infections are estimated to occur in the ocean each day, killing 20–40% of prokaryotes and releasing up to 109 tons of carbon from biological cells, significantly influencing ocean biogeochemical cycles [6]. Additionally, approximately 1014–1017 Gbp of DNA is estimated to be transduced by marine viruses daily, affecting host diversity and function [7]. Marine viruses have vast uncharacterized genetic diversity [8,9]. Although recent metagenomics studies have provided a huge amount of viral genetic information, most of them are considered as “dark matter” due to the lack of similarity with known sequences in the database. It is suggested that this problem could be partially addressed through the isolation and genetic characterization of novel culturable viruses, especially those isolated from deep-sea environments [10,11]. Thus, the isolation and characterization of novel viruses from the deep sea will help interpret the overwhelmingly unknown sequences of viral metagenomic data and improve our understanding of viral diversity and virus–host interactions in the deep sea.

*Bacillus tequilensis*, a gram-positive *Bacillus* first isolated from a sample taken from a roughly 2000-year-old shaft tomb near the city of Tequila, Jalisco, Mexico, was identified as a new subgroup of *Bacillus subtilis*, and is currently understudied [12]. Unlike some of their *Bacillus* relatives (e.g., *Bacillus anthracis*, *Bacillus cereus*), no pathogenic report of *B. tequilensis* has been published yet. *B. tequilensis* may have potential applications in biobleaching, exogenous bioremediation, food industry, textile dye decolorization, and plastic degradation, since several studies identified some industrial important enzymes from them, including alkaline protease [13], 1,3-1,4-β-glucanase [14], novel extracellular active thermo-alkali-stable laccase [15], alkaline pectate lyase [16], solvent-stable amylase and novel cellulases [17,18]. Moreover, *B. tequilensis* can use a carbon source to produce microbial exopolysaccharides, which have potential antioxidant activity [19]. Some *B. tequilensis* strains can produce biosurfactant lipopeptide, which has antibacterial and insecticidal activities [20,21]. So far, a total of 125 complete genomes of *Bacillus* phages have been deposited in the GenBank Database on NCBI (National Center for Biotechnology Information, https://www.ncbi.nlm.nih.gov/ (accessed on 22 February 2023)), with genome sizes ranging from 18 to 251 kb. These *Bacillus* phages infect a variety of *Bacillus* hosts; however, no phage infecting *B. tequilensis* has been reported yet. Since the potential importance of *B. tequilensis* in industrial applications, the isolation and characterization of novel phages infecting *B. tequilensis* is of great significance both for industry and phage biology. In this study, we isolated and characterized a novel *B. tequilensis*-infecting phage (designated as vB_BteM-A9Y) from deep-sea sediments in the South China Sea. To the best of our knowledge, vB_BteM-A9Y is the first phage reported to infect *B. tequilensis*.

## 2. Materials and Methods

### 2.1. Phage Isolation and Purification

*Bacillus tequilensis* KCTC 13622 strain was isolated from deep-sea sediments in the South China Sea. Phage plaques were observed during the culturing of *Bacillus tequilensis* KCTC 13622, which was grown at 25 °C in 2216E solid medium (0.01% ferric phosphate, 0.1% yeast extract, 0.5% tryptone, and 1.5% agar in seawater; pH 7.6). vB_BteM-A9Y phage particles were purified from phage-infected *B. tequilensis* KCTC 13622 using polyethylene glycol precipitation methods. In brief, a sterile loop was used to scrape a single plaque into 5 mL of liquid 2216E medium for overnight culturing at 25 °C, and then 5 mL of the overnight culture was inoculated into 400 mL fresh 2216E medium for expansion. After incubation, the culture was treated with 1.5 μg/mL DNase and RNase for 1 h at 25 °C; then 35 g sodium chloride was added, and the culture was placed on ice for more than 2 h. The phage-containing supernatant was collected after the culture was centrifuged at 15,000× *g* at 4 °C for 15 min. Subsequently, polyethylene glycol 8000 at a final concentration of 10% (*w*/*v*) was added to the supernatant and it was incubated at 4 °C for more than 12 h. The precipitated phages were collected by centrifugation at 15,000× *g* for 30 min at 4 °C and were resuspended in SM buffer (100 mM NaCl, 8 mM MgSO_4_, and 50 mM Tris-HCl; pH 7.5). The resulting phage concentrate was filtrated through a 0.22 μm filter and subjected to morphologic observation and DNA extraction.

### 2.2. Transmission Electron Microscopy (TEM) Observation

For TEM observation, one drop of purified phage particle solution was adsorbed onto the 200-mesh carbon-coated copper grid. After staining with 2% uranyl acetate for 1 min, samples were examined at 120 kV voltage with a JEOL JEM2100F TEM (JEOL, Tokyo, Japan).

### 2.3. One-Step Growth Curve

One-step growth curve analysis was conducted to characterize the infectivity and replication ability of vB_BteM-A9Y. Phages were mixed with exponentially growing *B. tequilensis* KCTC 13622 with a multiplicity of infection (MOI) of 0.01 and incubated at room temperature in the dark for 30 min to promote phage infections. Then, the cells were pelleted by centrifugation, re-suspended in 2216E medium, and diluted with fresh 2216E medium for 100 times to avoid a possible secondary infection. Thereafter, the cells were cultured at 25 °C with continuous shaking. Samples were collected at different time points between 0 h and 10 h post phage infection, and phage abundance in the supernatant was quantified using the double agar overlay plaque assay [22]. After the latent period, vB_BteM-A9Y underwent a single phage burst. The burst size, which indicates the average number of phage particles released per infected host cell, was calculated as the ratio between the number of plaque-forming units (PFUs) after and before the phage burst.

### 2.4. Phage Genomic DNA Extraction and Genome Sequencing

Prior to phage DNA extraction, the purified phage particles were treated with DNase I (Sangon Biotech, China) at 37 °C for 2 h to remove exogenous DNA fragments. Then, the phage particles were lysed with a combination of proteinase K (30 mg/mL, final concentration), SDS (1% *w*/*v*, final concentration), and EDTA (5 μM, final concentration) at 55 °C for 3 h. The lysate was then mixed with an equal volume of phenol/chloroform/isoamyl alcohol (*v*/*v* 25:24:1). The supernatant was collected by centrifugation at 15,000 *g* for 5 min and then sequentially purified by adding chloroform/isoamyl alcohol (*v*/*v* 24:1) and centrifugated at 15,000× *g* for 10 min. Subsequently, the supernatant was mixed with isoamyl alcohol to precipitate the DNA. The precipitate was then washed with cold 70% ethanol twice and then air-dried. Finally, the purified DNA was resuspended in TE buffer (10 mM Tris-HCl, 1 mM EDTA, pH 8.0) and stored at −20 °C until further analysis. The purified genomic DNA was sent to Hanyu Biotechnology Co., Ltd. (Shanghai, China) for whole-genome sequencing. A DNA library with an insert size of 300 bp was constructed using the NEBNext^®^ UltraTM DNA Library Prep Kit for Illumina (NEB, Ipswich, Massachusetts, USA). The phage genome DNA was sequenced by the Illumina Nova platform using the PE 2 × 150 bp strategy.

### 2.5. Phage Genome Assembly, Annotation, and Analysis

After high-throughput sequencing, Trimmomatic v0.32 [23] was used to remove low-quality reads and adapters. The obtained high-quality reads were assembled to phage genome sequence via velvet v1.2.03 [24], Newbler v2.8 [25]^,^ and SOAPdenovo2 v2.04 [26]. The PhageTerm software [27] was used to predict the termini of phage genome and DNA packaging mechanisms based on Next Generation Sequencing (NGS) data. Glimmer3 v3.02 [28], GeneMarkS v4.28 [29], and Prodigal v2.60 [30] were used for the prediction of open reading frames (ORFs). The ORF-encoded proteins were functionally annotated by searching against the NCBI NR, UniProt, and Pfam databases by using BLASTp and HHpred server [31,32]. The Virfam server was used to the recognition of head-neck-tail modules in phage genomes [33]. tRNA sequences were predicted using the tRNAscan-SE server [34]. The Viral Spacer database of the IMG/VR database was used to search for any putative protospacer in the genome of vB_BteM-A9Y (E-value < 10^−5^) [35].

For phage taxonomy, vConTACT2 v0.9.19 was used to compare the genome of vB_BteM-A9Y against the ProkaryoticViralRefSeq94 (v94) database with default parameters, and the phages predicted to be related with vB_BteM-A9Y were determined by similarity score > 1 [36]. Complete amino acid profiles of vB_BteM-A9Y and its related phages were submitted to the virus classification and tree building online resource (VICTOR) (https://ggdc.dsmz.de/victor.php, accessed on 16 February 2023) for phylogenetic analysis, and the recommended settings of the genome BLAST distance phylogeny (GBDP) method were used [37]. The proteomic tree of vB_BteM-A9Y and its related phages was generated using the ViPTree server [38] based on genome-wide sequence similarities computed by tBLASTx. The linear comparison of the genomes of vB_BteM-A9Y and its related phages was generated using the ViPTree server. Intergenomic nucleotide sequence similarity and aligned genome fractions within the imported phages were plotted with the Virus Intergenomic Distance Calculator (VIRIDIC) under recommended configurations [39]. The complete genome sequence of vB_BteM-A9Y is deposited in the GenBank database under the accession number ON528935.

## 3. Results and Discussion

### 3.1. Phage Isolation and Characterization

**Figure 1:**
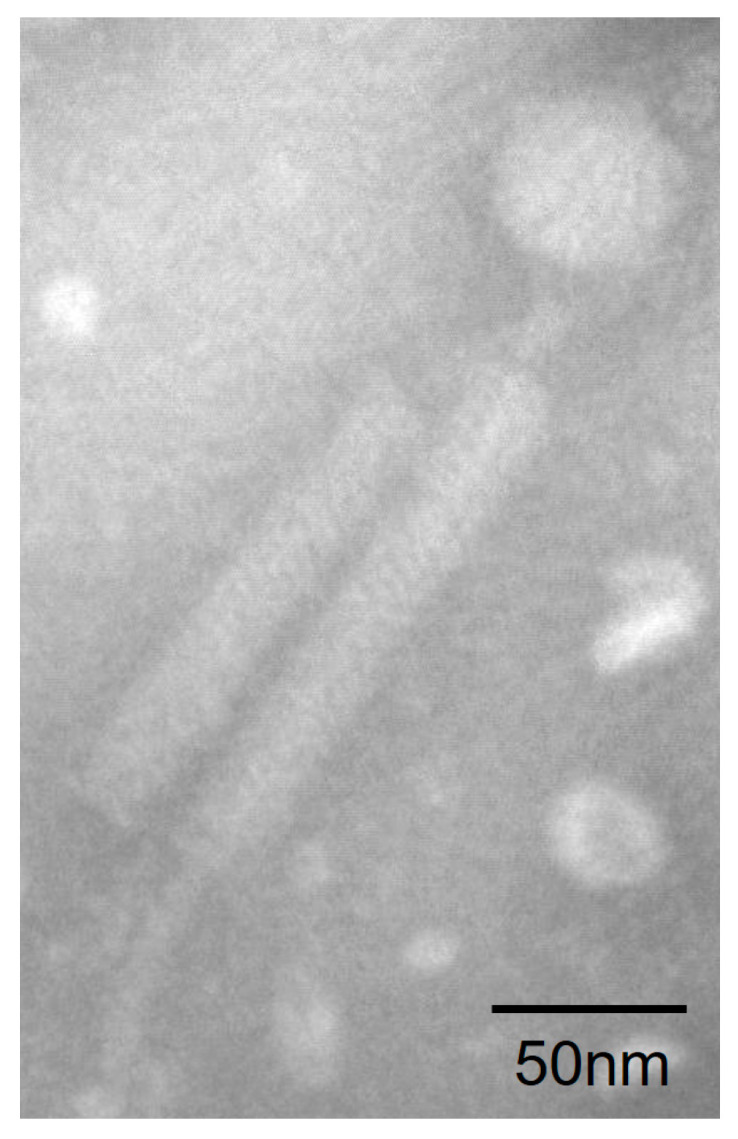
Morphology of vB_BteM-A9Y. Purified phage particles were negatively stained with 2% uranyl acetate on carbon-coated copper grids and observed using a JEM-1230 TEM at an accelerating voltage of 120 kV.

Plaques were found when the suspended deep-sea sediment from the South China Sea was spread over the 2216E culture plates. We purified the plaques and isolated phage particles by polyethylene glycol precipitation. The host shares 99.93% 16S rRNA similarity with *B. tequilensis*, and was further identified as *B. tequilensis* based on the genomic and biochemical characterizations. TEM observation showed that phage particles (designated as vB_BteM-A9Y) have the typical morphology of myophages. vB_BteM-A9Y has a long, complex, contractile tail consisting of a central tube surrounded by a contractile sheath and auxiliary structures, approximately 202.44 ± 3.95 nm in length and 23.34 ± 1.97 nm in diameter, and a hexamer head of an estimated diameter of 51.44 ± 2.58 nm (Figure 1).

**Figure 2:**
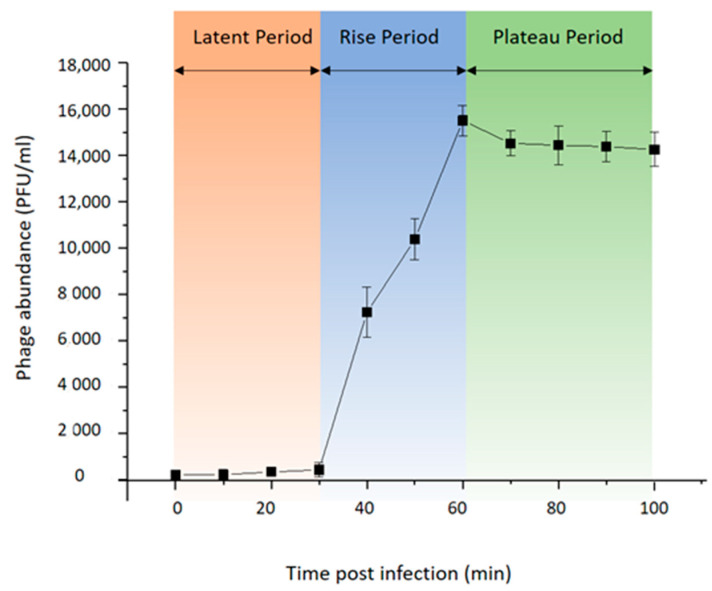
One-step growth curve of phage vB_BteM-A9Y. Each data point is shown as the mean ± SD of three independent replicates.

To investigate the life cycle of phage vB_BteM-A9Y, one-step growth curve analysis was conducted (Figure 2). Purified phage particles were used to infect the logarithmic growing host cells (OD_600_ = 0.2) at a MOI of 0.01. The result showed that vB_BteM-A9Y initiated host lysis at about 30 min post infection, and the titer of produced phage progenies reached a plateau at 60 min post infection. vB_BteM-A9Y exhibited a relatively small burst size of about 75 PFU per host cell.

### 3.2. Genomic Analysis of vB_BteM-A9Y

**Figure 3:**
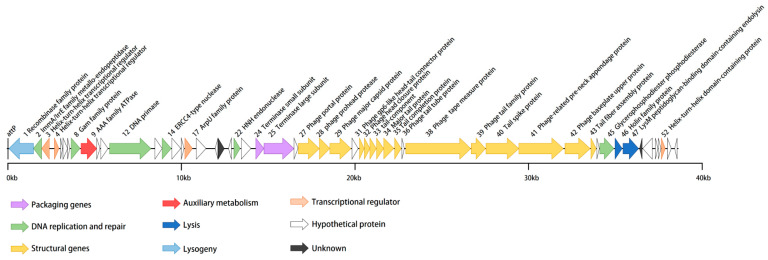
Annotated genome map of phage vB_BteM-A9Y. ORFs are depicted using leftward- or rightward-oriented arrows according to the predicted direction of transcription. ORF annotations are color-coded according to the legend below the map.

vB_BteM-A9Y has a linear double-stranded DNA genome with a size of 38,634 bp and a GC content of 41.05% (Table 1). The phage genome was predicted to encode a total of 54 ORFs with an average length of 656 bp. Similar to most bacteriophages, the genome of vB_BteM-A9Y is tightly arranged, with an average gene density of 1.397 genes/kb. Eight ORFs were transcribed leftward, and the remaining 46 ORFs were transcribed rightward (Figure 3). The translation products of all predicted ORFs were queried against the NCBI non-redundant RefSeq, UniProt, and Pfam databases by using BLASTp and HHrepd [31,32,40] (E-value cutoff = 10^−6^) to identify their putative functions. Only 27 of the ORFs were functionally annotated. No tRNA was found in the phage genome, implying that vB_BteM-A9Y is highly dependent on the host’s translation machinery. Moreover, no putative CRISPR protospacer was found in the genome. The genome termini were predicted based on NSG data using the software PhageTerm and the result indicated that vB_BteM-A9Y has unique obvious termini at both ends (R1 = 464 > 100, R2 = 57 > 3, and R3 = 73 > 3), suggesting that vB_BteM-A9Y employs a COS strategy for DNA packaging.

In the genomes of bacteriophages, ORFs encoding related biological functions tend to cluster, forming modules that can be co-regulated and co-inherited [41]. In the case of vB_BteM-A9Y, seven functional modules were identified in the genome, including modules involved in DNA packaging, structure morphogenesis, host lysis, DNA replication and repair, transcriptional regulation, lysogenic control, and auxiliary metabolism (Figure 3).

In the left arm (ORF1-ORF23) of the vB_BteM-A9Y genome, functionally related ORFs are dispersed and include ORFs predicted to be involved in DNA replication and repair, lysogenic control, transcriptional regulation, and auxiliary metabolism. A total of six ORFs in the vB_BteM-A9Y genome are predicted to participate in DNA replication and repair, including ImmA/IrrE family metallo-endopeptidase (ORF2), host nuclease inhibitor Gam family protein (ORF8), AAA family ATPase (ORF9), DNA primase (ORF12), ERCC4 type nuclease (ORF14), and HNH endonuclease (ORF22). ORF2 encodes a putative ImmA/IrrE family metallo-endopeptidase. IrrE is an important DNA repair regulatory protein that recognizes a wide range of DNA damage. It acts as a “universal switch” for DNA repair and protection pathways by regulating the expression of *recA* and *pprA*. The enhanced expression of *recA* and *pprA* stimulated by IrrE in response to ionizing radiation and UV light has been previously observed in *Deinococcus radiodurans* [42,43,44,45]. The putative host nuclease inhibitor Gam family protein (ORF8), first discovered and characterized from bacteriophage Mu, protects linear double-stranded DNA from exonuclease degradation in vitro and in vivo [46]. The putative DNA primase (ORF12) belongs to the P4 phage DNA primase family. This priming activity is similar to the enzymatic activity of DNA primases encoded by conjugative plasmids in terms of template utilization and the ability to synthesize primers [47]. ORF14 and 22 were predicted to encode the ERCC4-type nuclease and the HNH endonuclease, respectively. ERCC4-type DNA nuclease is a DNA repair nuclease; such enzymes are often part of the cellular response to UV-induced DNA damage [48,49]. HNH endonucleases have been identified in a number of bacteriophages and have been shown to play a variety of roles in the phage life cycle [50]; they comprise restriction, homing, and structure-specific endonucleases, as well as DNA repair-associated enzymes [51,52]. ORF9 encodes a putative AAA family ATPase. Phage-encoded AAA family ATPases are essentially the terminases that aid in the packaging of dsDNA in the procapsid during viral assembly, using ATP as an energy source [53]. However, AAA ATPases were also reported to have a putative role in the DNA recombination/repair/maintenance machinery in mycobacteriophages [54]. Given that ORF9 is clustered with ORFs involved in DNA repair and recombination, and both terminase large and small subunit genes have been identified in the vB_BteM-A9Y genome, it is likely that ORF9 participates in DNA repair and recombination. Collectively, the presence of ORF8, 9, 14, and 22 in the vB_BteM-A9Y genome suggests that vB_BteM-A9Y encodes multiple pathways for DNA damage repair, which may help to maintain the stability of the host/phage genome.

Several apparent gene clusters were identified in the middle and right arm (ORF24-ORF55) of the vB_BteM-A9Y genome, forming several functional modules involved in DNA packaging, structure morphogenesis, and host lysis. The DNA packaging module includes two genes, one coding for a terminase small subunit (ORF24) and the other encoding for a terminase large subunit (ORF25). ORFs encoding for capsid and tail morphogenesis were identified in the structure morphogenesis module. The capsid genes of bacteriophages are usually clustered in the order (in terms of transcription direction) of portal protein, proteases, scaffold proteins, and major capsid proteins [55]. Consistently, the capsid genes (ORF27-29) of vB_BteM-A9Y also follow this order, except that no separate scaffold protein was identified. The absence of a separate scaffold protein has also been observed in phage HK97. However, the N-terminal segment of the capsid protein (gp5) of phage HK97 contains a delta domain, which functions as a scaffold-like protein [56]. Given that the major capsid protein (ORF29) of vB_BteM-A9Y shares a low protein sequence similarity (27.78%) with its counterpart of HK97, it is unknown whether the capsid protein of vB_BteM-A9Y could have scaffold-like functions like its counterpart of phage HK97. Abundant ORFs encoding tail/neck structural proteins were identified (ORF31-43). The predicted gene product of ORF31 is highly homologous to the head-to-tail adaptor proteins of phage HK97 and phage SPP1, which serve as the interface for tail attachment and the point of egress for DNA from the head during infection [57,58,59]. Further Virfam server analysis showed that the neck module of vB_BteM-A9Y is classified into Neck Type 1-Cluster 2, which adopts the structural organization of the *siphovirus* SPP1 neck [33]. ORFs encoding for holin (ORF46) and endolysin (ORF47) were identified in the host lysis module, indicating that vB_BteM-A9Y achieves host lysis using a holin–endolysin system that is typical in dsDNA phages [60]. The predicted vB_BteM-A9Y endolysin contains a lysM peptidoglycan-binding motif, which shows the highest similarity with N-acetylmuramoyl-L-alanine amidase XlyA according to the results of HHpred server analysis and BLASTp analysis. LysM motif has been shown to be present in over 27,000 proteins and can bind to various types of peptidoglycan and chitin, in particular the N-acetylglucosamine moiety [61]; thus, it is likely that the endolysin of vB_BteM-A9Y degrades the host cell wall by recognizing N-acetylglucosamine moiety.

Four ORFs (ORF3, ORF4, ORF17, and ORF52) in the vB_BteM-A9Y genome were predicted to encode different types of transcriptional regulators. ORF48 is predicted to encode the ArpU family autolysin regulatory protein. It was previously reported that ArpU family transcriptional regulators act as transcriptional activators of late operons in Gram-positive bacteriophages, which could regulate phage DNA cleavage and packaging [62].

### 3.3. vB_BteM-A9Y Belongs to a Novel Genus under Caudoviricetes

**Figure 4:**
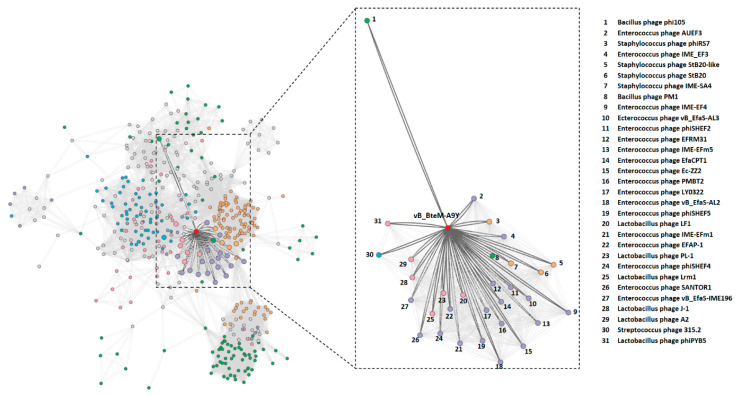
Protein-sharing network of vB_BteM-A9Y and 31 related phages with a pairing-similarity score >1. Each node represents the genome of a phage. Nodes (phages) are connected with edges when the pairing-similarity score >1 between phages, and the edges connecting vB_BteM-A9Y are displayed in bold and colored in dark gray. The subnet of vB_BteM-A9Y and its related phages are enlarged on the righthand side.

The BLASTn search of whole genome sequence of vB_BteM-A9Y against the GenBank revealed no existing homolog. Only two phage genomes show similarity to the vB_BteM-A9Y genome over low query coverage, i.e., Bacillus phage rho14 (GenBank accession number OM236514.1, 87.44% identity over 26% coverage) and Bacillus phage phi 105 (GenBank accession number NC_048631.1, 87.30% identity over 34% coverage). In addition, a total of 31 phages were predicted to be related with vB_BteM-A9Y (pairing-similarity score >1) by vConTACT2 analysis based on the protein-sharing network (Figure 4).

**Figure 5:**
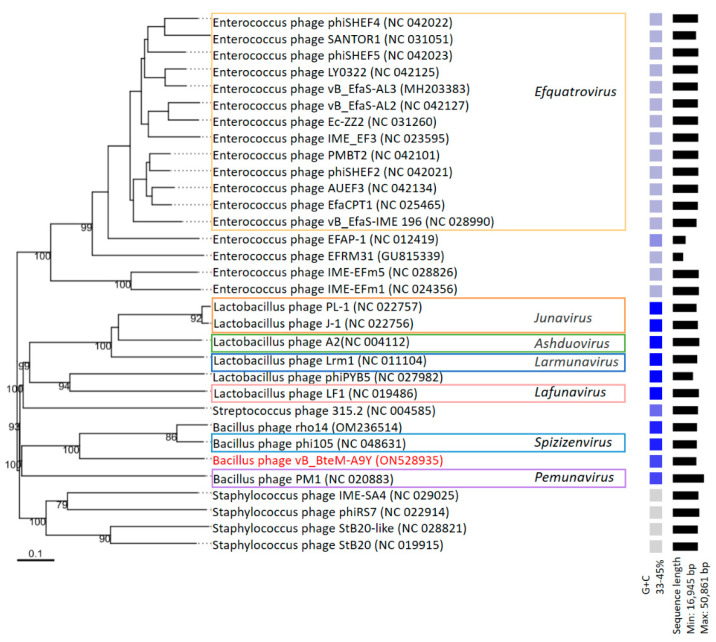
Phylogenomic tree of vB_BteM-A9Y and its related phages. This tree was generated using the Genome-BLAST distance phylogeny (GBDP) method, and the number near each node is the GBDP pseudo-bootstrap support value from 100 replications (only values > 50% are shown). Bacteriophage genus assignments according to the official ICTV classification (March 2023) are provided with different color frames. The GC content and sequence length of each phage genome is indicated on the right.

**Figure 6:**
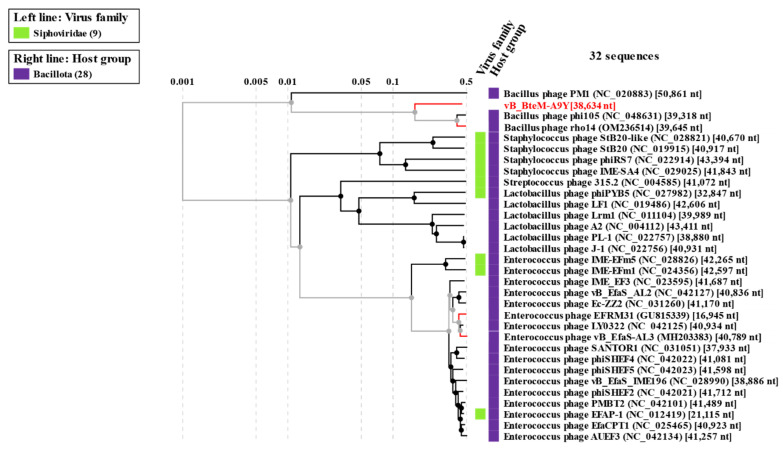
Phylogenetic analysis of vB_BteM-A9Y, Bacillus phage phi105, Bacillus phage rho14, and their most closely related phages based on whole-genome-wide sequence similarities calculated using tBLASTx.

A phylogenomic GBDP tree was constructed based on the whole-genome sequence of vB_BteM-A9Y and 31 related phages using the D6 formulas and yielding an average support of 30%. The results showed that vB_BteM-A9Y was clustered with Bacillus phage rho14 and Bacillus phage phi105 of the *Spizizenvirus* genus, with reliable bootstrap values but formed two deep branches (Figure 5). Consistently, the whole proteome-based phylogenetic tree showed the clustering of A9Y, rho14, and phi105 into two clades (Figure 6).

**Figure 7:**
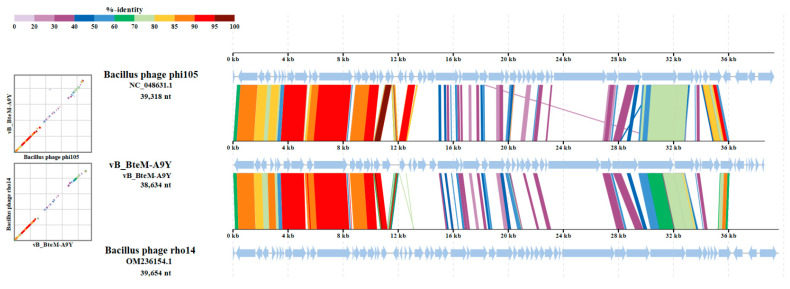
Comparative genomic organization of phage A9Y, Bacillus phage phi 105, and Bacillus phage rho14. The analysis was performed using ViPTree. The gradient scale indicates the similarity range between phage genomes.

**Figure 8:**
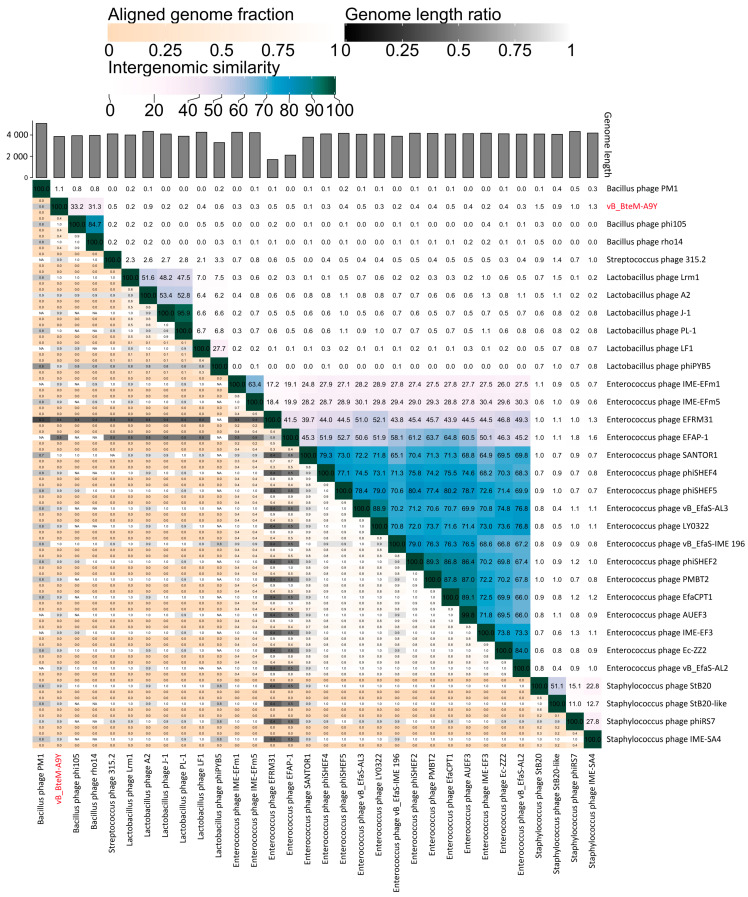
Intergenomic similarity between vB_BteM -A9Y and its related phages, calculated using VIRIDIC. The right half of this heatmap shows the similarity values between phage genomes. The left half of this heatmap shows the aligned genome fraction and genome length ratio.

A linear comparison of multiple loci between vB_BteM-A9Y and its most related phages (Bacillus phage phi105 and Bacillus phage rho14) was conducted. The results showed that the right arm of three phage genomes shows low homology, while the left arm of these genomes shows relatively high homology (Figure 7). Further comparative analysis of genomic nucleotide similarity and ORF homology of vB_BteM-A9Y with Bacillus phage rho14, Bacillus phage phi105, and 29 other related phages showed that the intergenomic similarity between vB_BteM-A9Y and these phages was 0–33.2% (Figure 8). According to the recognized virus classification standards published by ICTV, viruses in the same genus should share >50% similarity in nucleotide sequence or > 40% ORF homologs [63,64]. Collectively, based on the comprehensive morphological, phylogenetic, and comparative genomic analysis, we propose that vB_BteM-A9Y belongs to a novel genus under *Caudoviricetes*.

## 4. Conclusions

In this study, vB_BteM-A9Y, the first phage to infect *B. tequilensis*, was isolated from the deep-sea sediment of the South China Sea. Based on comprehensive morphological, one-step growth curve, phylogenetic, and comparative genomic analysis, we propose that vB_BteM-A9Y belongs to a novel genus under *Caudoviricetes*. Therefore, this study will increase our knowledge on deep-sea virus diversity and virus–host interactions, as well as expanding our knowledges on *Bacillus* phages.

## Figures and Tables

**Table 1:** Genomic characteristics of phage vB_BteM-A9Y.

	vB_BteM-A9Y
Genome length (bp)	38,634
GC content (%)	41.05%
Predicted ORF number	54
ORF total length (bp)	35,436
ORF length maximum (bp)	3777
ORF length minimum (bp)	111
ORF average length (bp)	656
ORF density (number/kb)	1.397
ORF/genome (%)	91.72
Predicted tRNA number	0
Predicted CRISPR protospacer number	0

## Data Availability

All data presented in this study are available in the manuscript text or deposited in the online database (GenBank, accession number ON528935).
